# Basal Cell Carcinoma of the Nipple: An Uncommon but Ever-Increasing Location

**DOI:** 10.1155/2011/818291

**Published:** 2012-01-12

**Authors:** Yasemin Oram, Cüyan Demirkesen, Ayşe Deniz Akkaya, Erkan Koyuncu

**Affiliations:** ^1^Department of Dermatology, American Hospital, Güzelbahçe Sokak No. 20, Nişantaşı, 34365 Istanbul, Turkey; ^2^Department of Pathology, Cerrahpasa Faculty of Medicine, Istanbul University, Fatih, 34098 Istanbul, Turkey

## Abstract

Basal cell carcinoma (BCC) is the most common malignancy of the skin. It is most frequently seen on the sun-exposed areas of the head and neck region. Occurrence of BCC on the nipple is extremely rare, though the number of the reported cases has been increasing steadily. It has metastatic potential to regional lymph nodes; therefore a more aggressive course can be expected when compared to BCCs located at other sites. Hence, early diagnosis and treatment of BCCs located on this region is of importance. There are 39 reported cases of BCC of nipple-areola complex (NAC) in the English literature. We present an additional case of BCC located on the nipple, presenting with enlargement of the nipple as a sole clinical finding in a 60-year-old man.

## 1. Introduction

Basal cell carcinoma (BCC) is the most common malignancy of the skin, and its incidence continues to increase worldwide. Fortunately in respect of the high incidence, primary BCCs rarely metastasize, with a rate of less than 0.1% [1]. BCCs most commonly arise from sun-exposed areas. BCC occurring on the nipple-areola complex (NAC) is extremely rare, and it has been known to have a higher metastatic potential than BCCs at other sites, showing a more aggressive course [2–4]. There are 39 reported cases of BCCs of the NAC in the English literature in the last 118 years. [2–8]. The number of the reported cases has almost been doubled in the past decade, and the incidence of BCC at this particular site appears to be ever increasing. Here, we report an additional case of primary BCC on the nipple.

## 2. Case Report

**Figure 1:**
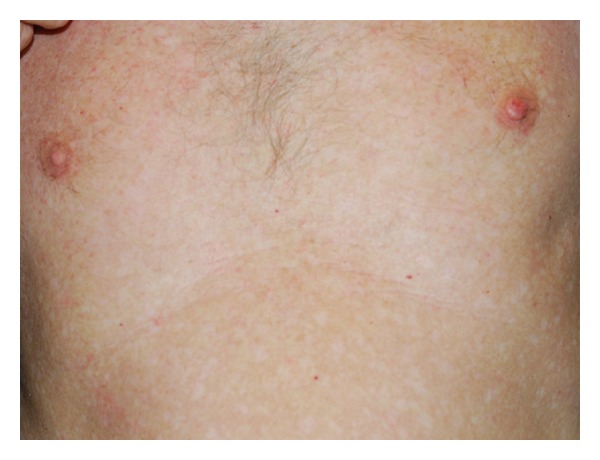
Enlarged left nipple. Clinical appearance of BCC on the left nipple. Erosion on the surface represents the biopsy site.

**Figure 2:**
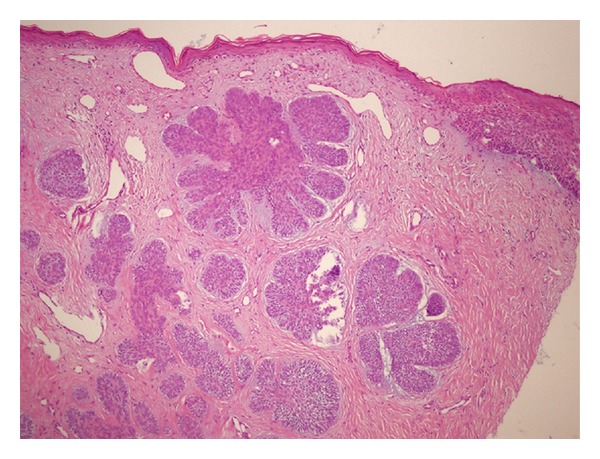
Basal cell carcinoma. Small islands of basaloid cells with peripheral palisading, showing connections with the overlying epidermis (HEX100).

A 60-year-old light-skinned male presented with a history of slow enlargement of his left nipple. The patient had been examined 4 years ago and a slight asymmetry of the nipples had been noticed. He had been referred to the general surgery clinic for further evaluation of the asymmetry of the nipples. Clinical examination of the general surgeon and the mammography had revealed no significant findings. He had been under followup on a regular basis. After 4 years, at his subsequent admittance to the dermatology clinic, on dermatologic examination, the left nipple was firm and larger than the right one, measuring 1.0 cm × 0.8 cm. The areola was uninvolved (Figure 1). There was no bleeding or ulceration of the lesion, and there was no axillary lymphadenopathy. His relevant past medical history included 6 previous BCCs, 2 of which were located over the trunk, and multiple actinic keratosis. He had history of severe sun exposure. He was healthy otherwise. A punch biopsy from the left nipple was performed and the histopathological examination revealed a mixed histological pattern of BCC composed of superficial and micronodular types (Figure 2). The patient was then referred to the plastic surgery clinic for a large excision and sentinel lymph node biopsy. The patient refused to have an extensive surgery and was lost to followup.

## 3. Discussion

Skin cancers are the most common type of cancer accounting for about half of all human cancers. The most common type of skin cancers are nonmelanoma skin cancers, with BCCs representing 70–80% of all cases [9]. Although the etiology of BCC is multifactorial, ultraviolet radiation has been considered the most important triggering factor [1]. Hence, BCCs most frequently arise from the sun-exposed areas of the head and neck region [10]. As the NAC is a sun-protected area, the occurrence of BCC on this site is quite rare with a male predominance in the reported cases. Thirty-nine cases of BCC of the NAC were identified in the English literature in the last 118 years [2–8]. Twenty-five of the reported cases were male and only one-third of the patients presented solely with the nipple involvement. The male preponderance is possibly due to the increased exposure of the chest area to sunlight in males [8], although male-to-female ratio is similar to that for BCCs of more common sites [4].

1. Benharroch and colleagues have postulated that the number of reported cases does not reflect the actual incidence of BCCs at this site, as not each case is reported or can be published [2, 11]. The literature search revealed that the number of reported cases of BCCs on the NAC has almost been doubled in the past decade. This can be attributed in part to increased awareness and detection of skin cancers in general [9]. Moreover, the increased number of similar cases might have led the physicians to agonize BCCs at this uncommon site.

The present case was a 60-year-old male who had BCC on the nipple with a sole clinical finding of slow enlargement of the nipple over the years. The clinical differential diagnosis of BCCs of NAC includes Paget's disease, Bowen's disease, erosive adenomatosis, and contact dermatitis when there are erythematous, scaling, or ulcerating lesions [11]. The chief clinical findings in the previously reported cases were plaques and papules, masses, erythema, oozing, exudation, edema, and eczema [4]. However, the presentation of BCC in our case is very unusual that the BCC resides in the nipple, resembling a firm nipple. This presentation can easily be misdiagnosed, particularly when it is small or the asymmetry is not very prominent. Mammographic findings of BCCs on the NAC may consist of microcalcification and involution, or more often there is no finding at all [4, 5]. The present case also underwent mammography, revealing no significant findings.

Although BCCs can become locally aggressive and cause extensive tissue destruction, fortunately metastatic rate of a primary BCC is less than 0.1% [1]. However BCCs located on the NAC have been considered to be more aggressive. Ferguson et al. have reported a metastatic rate of BCCs over NAC as 9.1% in a retrospective literature search [2]. In another literature review, Takeno et al. have reported a metastatic rate of 11.5% of BCCs arising from the NAC [4]. The reported BCCs that metastasized were large and ulcerated. The higher potential of metastasis for tumors in this location is possibly due to the rich network of lymphatic capillaries in the subareolar plexus that provides an easy route for tumor spread [2, 12]. Therefore the treatment of BCCs located at the NAC requires special attention. Previously reported cases underwent surgical treatments including simple mastectomy, mastectomy with axillary lymph node dissection, irradiation or sentinel node biopsy, partial mastectomy, and Mohs surgery. Taking the rich lymphatic flow in the NAC and relatively high incidence of lymph node metastasis into consideration, sentinel node navigation surgery should be considered together with the local resection as a part of the surgical treatment [4]. The presented case did not accept to have large excisional surgery and sentinel lymph node biopsy.

Although BCC of the NAC is extremely rare, its incidence is ever increasing. Moreover, BCCs over NAC can be potentially aggressive and can result in metastasis. Therefore it appears to be of great importance to recognize the BCCs at this site, giving consideration that unusual presentations could emerge at unusual sites, particularly in older individuals with light complexions, severe sun damage, and previous history of BCCs and actinic keratosis.
